# Prescribing and deprescribing in older people with life-limiting illnesses receiving hospice care at the end of life: A longitudinal, retrospective cohort study

**DOI:** 10.1177/02692163231209024

**Published:** 2023-11-30

**Authors:** Tahani Alwidyan, Noleen K McCorry, Chris Black, Rachel Coulter, June Forbes, Carole Parsons

**Affiliations:** 1 Department of Clinical Pharmacy and Pharmacy Practice, Faculty of Pharmaceutical Sciences, The Hashemite University, Zarqa, Jordan; 2 School of Medicine, Dentistry and Biomedical Sciences, Queen’s University Belfast, Belfast, Northern Ireland, UK; 3 Marie Curie Hospice, Belfast, Antrim, UK; 4 Foyle Hospice, Derry/Londonderry, Northern Ireland, UK; 5 Northern Ireland Hospice, Belfast, Northern Ireland, UK; 6 School of Pharmacy, Queen’s University Belfast, Belfast, UK

**Keywords:** Prescribing, deprescribing, inappropriate prescribing, older people, end of life, hospice care, medicines optimisation

## Abstract

**Background:**

Although prescribing and deprescribing practices in older people have been the subject of much research generally, there are limited data in older people at the end of life. This highlights the need for research to determine prescribing and deprescribing patterns, as a first step to facilitate guideline development for medicines optimisation in this vulnerable population.

**Aims:**

To examine prescribing and deprescribing patterns in older people at the end of life and to determine the prevalence of potentially inappropriate medication use.

**Design:**

A longitudinal, retrospective cohort study where medical records of eligible participants were reviewed, and data extracted. Medication appropriateness was assessed using two sets of consensus-based criteria; the STOPPFrail criteria and criteria developed by Morin et al.

**Setting/participants:**

Decedents aged 65 years and older admitted continuously for at least 14 days before death to three inpatient hospice units across Northern Ireland, who died between 1st January and 31st December 2018, and who had a known diagnosis, known cause of death and prescription data. Unexpected/sudden deaths were excluded.

**Results:**

Polypharmacy was reported to be continued until death in 96.2% of 106 decedents (mean age of 75.6 years). Most patients received at least one potentially inappropriate medication at the end of life according to the STOPPFrail and the criteria developed by Morin et al. (57.5 and 69.8% respectively). Limited prevalence of proactive deprescribing interventions was observed.

**Conclusions:**

In the absence of systematic rationalisation of drug treatments, a substantial proportion of older patients continued to receive potentially inappropriate medication until death.

**What is already known about the topic?**

- Polypharmacy and potentially inappropriate prescribing are prevalent in older people in general.
- Chronic medications are of limited benefit to older people with life-limiting illnesses at the end of life where the treatment goal shifts towards symptom control rather than disease cure.
- One potential solution to improve prescribing appropriateness among this vulnerable population is deprescribing, a supervised discontinuation of potentially inappropriate medicines.

**What this paper adds?**

- This study shows that many older people received at least one medication of questionable clinical benefit or potentially inappropriate medication during the last two weeks of life.
- In the hospice setting, deprescribing was often reactive rather than proactive in routine clinical practice.
- Higher numbers of potentially inappropriate medications were identified when the two prescribing appropriateness tools were applied concurrently for the same patient than when each set of criteria was used separately.

**Implications for practice, theory or policy**

- Routine adoption of deprescribing tools into clinical practice should guide treatment management decisions.
- Investigation of the practicality of concurrent application of two sets of prescribing appropriateness criteria in routine clinical practice is warranted in terms of time and resource.
- Implementation of a proactive deprescribing intervention could help in reducing inappropriate polypharmacy to a significant extent.
- There is an imperative need for deprescribing guidance, educational sessions, and training workshops which could assist clinicians to rationalise medicines use in this vulnerable population.

## Introduction

Polypharmacy is common in older people and increases the risk of potentially inappropriate prescribing,^1^ defined as the use of medications where their harm outweighs their therapeutic benefit.^1^ In older people at the end of life, the prescription of potentially inappropriate medications is prevalent and continues as death approaches.^2–7^ Until recently, no criterion-based tools have been available to assist identification of potentially inappropriate prescribing in people with limited life expectancy. However, recent years have seen the development of tools specifically targeted at appropriateness of prescribing for this vulnerable population, including the STOPPFrail criteria,^8,9^ and the criteria developed by Morin et al.^10^ There is a growing evidence-base for STOPPFrail in identifying and reducing potentially inappropriate medications^5,11–13^ whereas the identification of potentially inappropriate medications using the criteria developed by Morin et al. is more limited.^7^ No previous study has compared the use of the STOPPFrail criteria to other prescribing appropriateness tools in older people with life-limiting illness at the end of life in hospice care.

Deprescribing,^5^ a supervised and structured process of medication discontinuation, has been suggested as an approach which improves health outcomes.^14^ In recent years, a distinction has been made between proactive and reactive deprescribing in older people.^15^ Proactive deprescribing describes the rational discontinuation of medications to prevent future harm, whereas reactive deprescribing refers to medication discontinuation in response to adverse clinical triggers.^15^ While the number of studies investigating deprescribing patterns^16^ and clinically significant outcomes of deprescribing in older people at the end of life are limited,^17–19^ deprescribing has been associated with positive effects on mortality, and in reducing falls^20^ and potentially inappropriate medications in older people at the end of life.^20,21^ Admission to an inpatient hospice unit and the consequent close monitoring of patients should facilitate proactive deprescribing.^22^ However, patterns of deprescribing in the hospice setting are poorly understood and there is a need to identify the extent to which it currently occurs in clinical practice.^23^ This study aimed to quantify patterns of prescribing and deprescribing and to examine the differences in the prevalence of potentially inappropriate medications in older people at the end of life in inpatient hospice units according to the STOPPFrail criteria^8^ and the criteria developed by Morin et al.^10^

## Methods

### Ethical approvals

Ethical approval was obtained from the Health and Social Care Research Ethics Committee B (HSC REC B) (reference 19/NI/0114) on 5th July 2019. Approvals were also secured from the ethics/governance committees of participating hospices. Data were extracted retrospectively from the records of deceased patients; it was not possible to obtain consent.

### Design

This longitudinal, retrospective cohort study consisted of two phases; firstly, patients’ medical records were reviewed and data extracted. Secondly, the STOPPFrail criteria^8^ and the criteria developed by Morin et al.^10^ were applied to the prescribing data.

### Setting

Data for this study were collected from three hospices spread geographically across Northern Ireland. These hospices provide pain and symptom control, and psychological and social support for terminally ill patients through inpatient, day therapy and community services settings. At the time of this study, there was no accepted time frame (i.e., estimated life expectancy) for hospice care eligibility in Northern Ireland.

### Participants

In the United Kingdom, most hospices offer inpatient admission for symptom control when persons are recognised to be in the last 2 weeks of life.^24^ Therefore, the last 14 days of life were determined to be end of life in this study. All decedents aged 65 years and older admitted to participating hospices continuously for at least 14 days before death who died between 1st January and 31st December 2018 and who had a known diagnosis, known cause of death, and prescription data were eligible and included in the study. Exclusion criteria were sudden, unexpected or unanticipated death as determined from medical records (e.g. cardiac arrest, pulmonary embolism or sepsis).

### Data extraction

A data extraction proforma was developed and refined with assistance and feedback from palliative care specialists in hospice care. In each hospice, a healthcare professional who had a direct care relationship with patients identified eligible decedents’ records and assigned a unique code to each patient. Data were extracted and pseudo-anonymised between October 2019 and January 2021 by the healthcare professional from each participating hospice, before being passed on to the researcher (TW).

Medications were grouped into symptom control medications, chronic medications, and antimicrobial agents. All medications recommended for symptom control in the Palliative Care Pain and Symptom Control Guidelines for Adults^25^ were considered as symptom control medications. The term ‘chronic medication’ refers to medications for treating chronic medical conditions, malignant diseases, or preventing medical conditions.^26–28^ In circumstances where an indication for a medication was not documented, medication classification was determined based on the patient’s medical history and/or on the known indication in literature by the researcher TW and the healthcare professional in the participating hospice. Medications were categorised according to their pharmacological class using the Anatomical Therapeutic Chemical code.

Due to the lack of universal consensus on the definition of polypharmacy,^29–31^ polypharmacy was defined as the concomitant use of five to nine regular medications, while excessive polypharmacy was defined as the concomitant use of ten or more regular medications^32^ for the purposes of this study. These definitions have been used previously in other population-based studies.^33–35^ Prescribed medications were those administered on a regular basis during the last 14 days of life, either continued or initiated during hospice admission. Over-the-counter and ‘when required’ medications were not included.

### Medication review for appropriateness

Prescribed medications during the last 14 days of life were reviewed for the prevalence of potentially inappropriate medications using the STOPPFrail criteria, and the criteria developed by Morin et al. for drug continuation/initiation. It was only possible to apply 20 of the 25 STOPPFrail criteria for some patients for whom the dates of prescription for proton pump inhibitors (PPIs), H_2_ receptor antagonists, neuroleptic antipsychotics, long-term oral non-steroidal anti-inflammatory drugs (NSAIDs), and long-term oral steroids were unavailable. It was not possible to apply criteria D1, E1, E2, G4 and G5^8^ which consider duration of treatment in determination of mediation appropriateness.^8^ Although the original published criteria^10^ for use of the criteria developed by Morin et al. included: older adults (⩾75 years); and an estimated life expectancy of 3 months or less, the authors confirmed (personal communication from corresponding author) that the tool can be applied to patients aged 65 years and older. Furthermore, other researchers have applied this tool in older people aged 50 years and over in a previous study.^36^

### Deprescribing patterns

Depending on the documented reason for discontinuation in decedents’ medical files, medication discontinuations were classified into reactive deprescribing or proactive deprescribing.^15^ In some circumstances, the stopping of medication was determined not to constitute deprescribing (e.g. change in the route of administration).

### Statistical analysis

Statistical Package for the Social Sciences (SPSS®) V26.0 (IBM, New York, USA) was used for statistical analysis. Descriptive analyses were used to calculate mean age, mean number of comorbidities and mean number of medications prescribed and deprescribed. For continuous variables, the Shapiro-Wilk test was used to test the normality of distribution. Parametric paired *t*-tests and non-parametric Wilcoxon tests were used to analyse the mean differences between medication numbers prescribed from day 14 before death to the day of death. Significance was set *a priori* at *p* ⩽ 0.05.

## Results

### Demographic and clinical characteristics of study cohort

A total of 106 decedents with a mean age of 75.6 (±7.2) years were included in this study; 63.2% were female. Decedents had a mean length of hospice stay of 30.9 (±17.6, [range 14–108]) days before death and a mean number of 3.9 (±1.6, [range 1–8]) chronic medical conditions. For most decedents, cancer was the reason for hospice admission (88.7%) and the most common cause of death (92.5%). By the day of death, 41 (38.7%) decedents were prescribed between 5 to 9 medications (polypharmacy) and 61 (57.5%) decedents were prescribed 10 or more medications (excessive polypharmacy) (Table 1).

**Table 1.** Demographic and clinical characteristics of study cohort.

Sample size	106 (100)
Age categories (years)	
65–69	23 (21.7)
70–74	29 (27.4)
75–79	25 (23.6)
80–84	17 (16)
85–89	6 (5.7)
⩾90	6 (5.7)
Gender	
Female	67 (63.2)
Male	39 (36.8)
Place of residence prior to admission	
Patient’s own home	53 (50)
Hospital	49 (46.2)
Other	4 (3.8)
Length of hospice stay	
Mean (±SD, [range])	30.9 (±17.6, [14–108])
Number of chronic medical conditions	
Mean (±SD, [range])	3.9 (±1.6, [1–8])
Reason for hospice admission	
Cancer	94 (88.7)
Symptom control	3 (2.8)
Not documented	2 (1.9)
COPD	2 (1.9)
Pulmonary fibrosis	1 (0.94)
Motor neurone disease	1 (0.94)
Multi-system atrophy	1 (0.94)
Parkinson’s disease	1 (0.94)
Pneumonia	1 (0.94)
Cause of death	
Cancer	98 (92.5)
COPD	2 (1.9)
Pneumonia	1 (0.94)
Parkinson’s disease	1 (0.94)
Pulmonary fibrosis	1 (0.94)
Respiratory failure	1 (0.94)
End stage renal disease	1 (0.94)
Motor neurone disease	1 (0.94)
Number of medications prescribed per patient on day 14 before death	
Less than 5	2 (1.9)
5–9	11 (10.4)
10 or more	93 (87.7)
Number of medications prescribed per patient on day 7 before death	
Less than 5	1 (0.9)
5–9	15 (14.2)
10 or more	90 (84.9)
Number of medications prescribed per patient on the day of death	
Less than 5	4 (3.8)
5–9	41 (38.7)
10 or more	61 (57.5)

COPD: chronic obstructive pulmonary disease; SD: standard deviation.

### Prescription pattern

**Figure 1.**
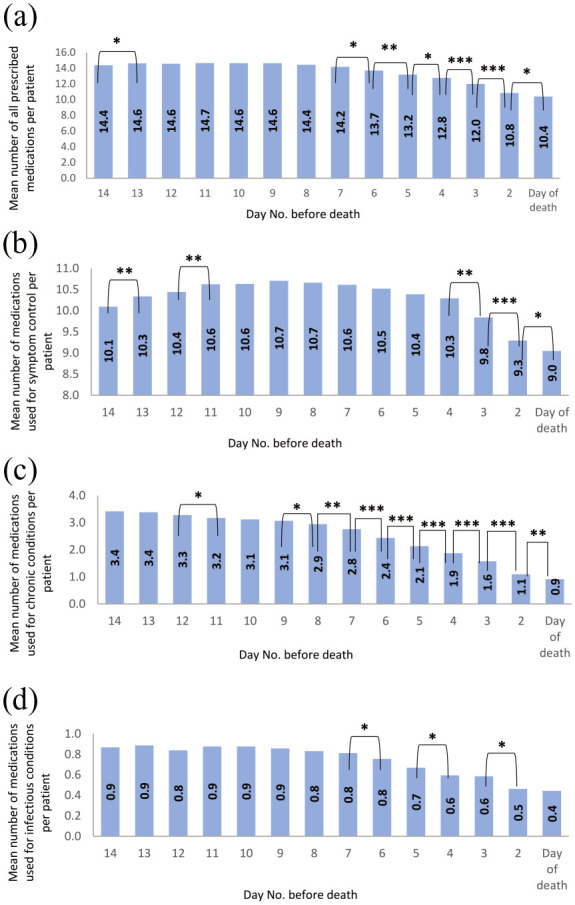
Mean number of medications prescribed during the last 14 days of life: (a) mean number of all prescribed medications, (b) mean number of medications used for symptom control, (c) mean number of medications used for chronic conditions, and (d) mean number of medications used for infectious conditions. *p ⩽ 0.05. **p ⩽ 0.01. ***p ⩽ 0.001.

During the last 14 days of life, 1897 different medications were prescribed for all decedents (mean 17.9 ± 4.8 medications), the majority of which (1354, 71.4%) were prescribed for symptom control. Lower proportions of medications (394 (20.8%) and 149 (7.9%)) were prescribed to treat chronic conditions and infections, respectively. The mean number of medications prescribed per patient decreased significantly between day 14 and the day of death, from 14.4 ± 4.4 medications to 10.4 ± 3.9 medications (*p* < 0.001). There was a significant increase in the mean number of symptom control medications between day 14 and day 8 before death from 10.1 ± 3.7 medications to 10.7 ± 3.4 medications (*p* = 0.003). However, in the last 7 days of life, a steady decrease was observed in the mean number of all medication classes. By the day of death, the mean number of chronic medications reduced significantly to approximately 25% (from 3.4 on day 14 before death to 0.9 on the day of death (*p* < 0.001)), while the mean number of medications reduced significantly by nearly half and just over 1/10th for antimicrobial agents and symptom control medications, respectively (*p* < 0.001) (Figure 1).

### Prevalence of potentially inappropriate prescribing

**Figure 2.**
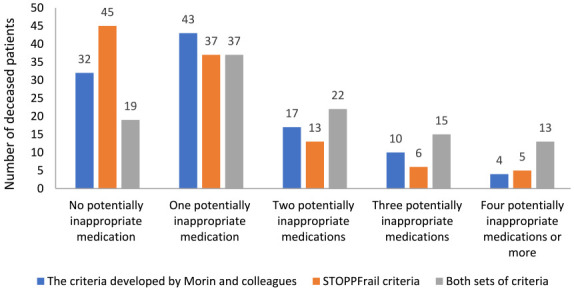
Numbers of patients with potentially inappropriate medication(s) according to the criteria developed by Morin et al., the STOPPFrail and both sets of criteria.

According to STOPPFrail, just over half (57.5%) of 106 decedents received at least one potentially inappropriate medication. A higher proportion (69.8%) received at least one potentially inappropriate medication according to the criteria developed by Morin et al. (questionable (62.3%) or often inadequate (18.8%) clinical benefit). Using both sets of criteria concurrently for the same patient resulted in a statistically significant increase in the number of patients determined to be receiving one or more potentially inappropriate medication in the last 14 days of life compared to using either set alone (82.1% of 106 decedents, *p* < 0.001) (Figure 2). Among those decedents, a statistically significant difference was seen between the number of potentially inappropriate medications identified using STOPPFrail and the criteria developed by Morin et al. (*p* = 0.046); there were 102 and 123 potentially inappropriate medications identified using these criteria, respectively. Over half (55.3%) of the potentially inappropriate medications identified by the criteria developed by Morin et al., were initiated during hospice admission. Gastrointestinal and cardiovascular system medications were the most commonly implicated medication classes identified using the two sets of criteria (Tables 2 and 3).

**Table 2.** Number (%) of potentially inappropriate medications within pharmacological classes according to STOPPFrail (*n* = 102).

Medication class	*n* (%)
Gastrointestinal system	21 (20)
Cardiovascular system	20 (19)
Endocrine system	18 (18)
Musculoskeletal system drugs	12 (12)
Nutrition supplements	10 (10)
Coagulation system	7 (7)
Urogenital system	5 (5)
Multivitamin combination supplements	4 (4)
Folic acid	3 (3)
Respiratory system	2 (2)

**Table 3.** Number (%) of continued and initiated potentially inappropriate medications within pharmacological classes according to criteria developed by Morin et al. (*n* = 123): (a) number (%) of potentially inappropriate medications of questionable clinical benefit and (b) number (%) of potentially inappropriate medications of often inadequate clinical benefit.

(a) Questionable clinical benefit			
Continued medication class	*n* (%)	Initiated medication class	*n* (%)
Cardiovascular system	13 (10.6)	Gastrointestinal system	22 (17.9)
Coagulation system	12 (9.8)	Antidepressant	11 (8.9)
Endocrine system	6 (4.9)	Endocrine system	9 (7.3)
Drugs used in malignant disease	5 (4.1)	Coagulation system	8 (6.5)
Gastrointestinal system	3 (2.4)	Cardiology system	3 (2.4)
Urogenital system	2 (1.6)	Urogenital system	1 (0.8)
Respiratory system	2 (1.6)	Anti-Parkinson drugs	1 (0.8)
(b) Often inadequate clinical benefit			
Continued medication class	*n* (%)	Initiated medication class	*n* (%)
Vitamins and minerals	7 (5.7)	Vitamin and minerals	11 (8.9)
Acetylcholinesterase inhibitors	3 (2.4)	Antidepressant	1 (0.8)
Nutrition, blood products and electrolytes	1 (0.8)	Cardiology system	1 (0.8)
Musculoskeletal system drugs	1 (0.8)		

### Deprescribing patterns

**Figure 3.**
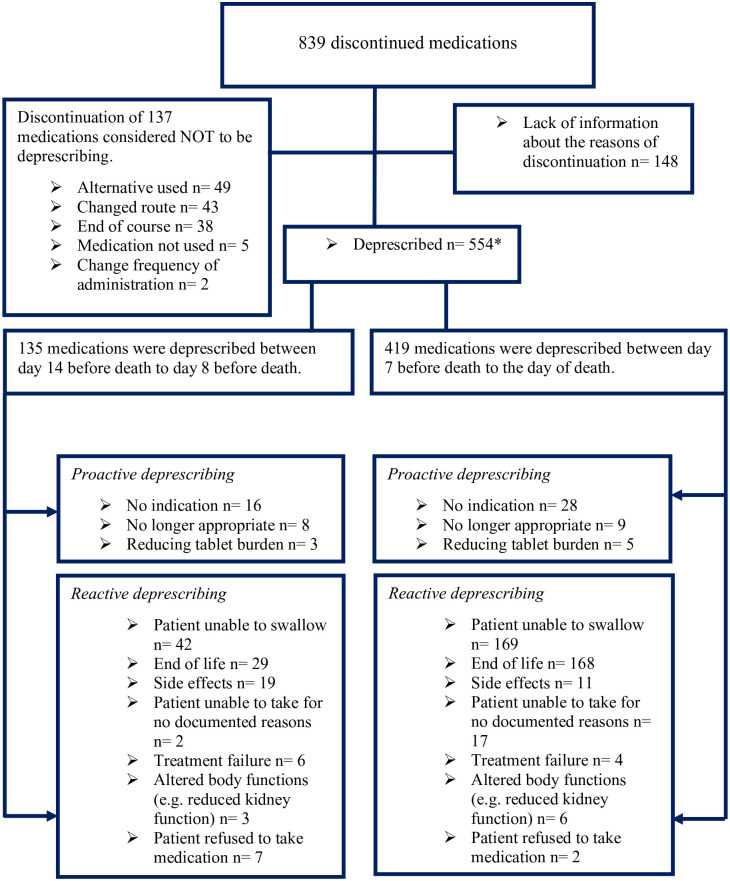
Categorisation of discontinued medications. *2.8% of the 554 medications deprescribed were documented as stopped by weaning.

Among 106 decedents, 839 medications were discontinued during the last 14 days of life. According to the documented reason in patients’ medical notes, 554 medicines (64.2%) were considered to have been deprescribed; of these, 12.5% were considered to be proactive deprescribing. Recognition of swallowing problems was the main reason for reactive deprescribing in the last 14 days of life, followed by end-of-life prognosis. Not all medications recorded as discontinued are deprescribed (for example, where the reason is a change in the route of administration); this was identified for 137 medications. It was not possible to categorise the discontinuation of 148 medications due to the lack of information about the reasons for discontinuation (Figure 3).

## Discussion

### Main findings

This study is the first to differentiate between reactive and proactive deprescribing in older people at the end of life in hospice care. While limited proactive deprescribing activity was identified, prescribers reactively deprescribed medications when clinical triggers such as swallowing problems and imminent end-of-life status were recognised among this vulnerable population.

The findings of this study suggest that deprescribing for older people at the end of life in hospice care relies particularly on assessing the potential for harm (87.5% of discontinuations were reactive deprescribing) rather than the evaluation of risks and benefits (12.5% of discontinuations were proactive deprescribing). There were only three documented reasons which guided the proactive deprescribing process: medication with no indication, no longer appropriate medication and reducing tablet burden. In the present study, the significant proportion of decedents who continued to receive potentially inappropriate medications until death may be linked to the observed limited adoption of pre-emptive (proactive) deprescribing. There may therefore be opportunity for increasing proactive deprescribing activity in hospice care. However, the extent to which this is feasible and acceptable is still unknown. Future research should therefore seek to explore the barriers and enablers for prescribers and patients to increase proactive deprescribing activity in hospice care.

### Interpretation and implications for clinical practice

The lack of data about medication prescribing in older people at the end of life is known in literature.^37^ In line with previous studies,^38,39^ this study reported a significant decrease in the mean number of medications from 2 weeks before death to the day of death. However, almost all decedents continued to experience polypharmacy or excessive polypharmacy at the day of death. This study reported that prescribing of symptom control medications increased between days 14 and 8 before death but decreased in the last week of life. A similar trend was observed in a retrospective cohort study among hospice patients in Japan.^40^ A continuous decrease in the mean number of chronic medications was observed in the current study from day 14 before death to the day of death. Albeit in a different healthcare setting, this is consistent with the findings of a retrospective cohort study of older nursing home residents in Australia.^39^

The availability of tools that systematically assess medication appropriateness and their application as part of medicines optimisation is growing in the literature.^4,12,41–43^ One of these studies^12^ reported that PPIs, calcium supplements, lipid-lowering agents and antipsychotics were the most prevalent classes of potentially inappropriate medications using STOPPFrail. Contrary to these findings, none of the decedents in the present study received lipid-lowering agents during the last 2 weeks of life and potentially inappropriate medications did not include inappropriate use of antipsychotics. This may be attributed to the close proximity to death for the patients included in the present study. Despite the differences in the conditions causing limited life expectancy and the heterogeneity of healthcare settings, the findings of two separate longitudinal, retrospective cohort studies^4,43^ and those of the present study all confirm that medication of questionable or of often inadequate clinical benefit are prevalent in older people at the end of life according to the criteria developed by Morin et al. Since cancer was the reason for hospice admission among most of our study sample, it should be acknowledged that more specific tools to assess medication appropriateness in cancer patients in general^44,45^ or in cancer patients who are receiving palliative care^46^ are available. The present study included all patients in hospice care, regardless of the terminal illness diagnosis.

Since there are differences in the number of potentially inappropriate medications identified in this study using the two sets of criteria, adjuvant use of both could be valuable for prescribers in considering potentially inappropriate medications. The proportion of decedents receiving at least one potentially inappropriate medication exceeded 80% when both sets of criteria were applied concurrently for the same patient. Albeit not in the end-of-life context, the use of multiple criteria in assessing the prevalence of potentially inappropriate medications in the same population of older people is reported in the literature.^47–49^ A prospective observational study conducted using Beers and STOPP criteria concurrently in hospitalised patients in Italy^50^ reported that the combination of the two tools could identify larger proportions of older patients receiving potentially inappropriate medications than applying a single set of criteria.^50^

While reactive deprescribing is important in management of clinical issues such as inability to swallow, prevention of harm should be prioritised through adoption of proactive deprescribing in clinical practice, through routine review and rationalisation of the risks and benefits of medications.^51^ One UK study in the hospital setting reported the discontinuation of 200 medications across 415 older people. Of these, 44 (22.0%) were confirmed as deprescribing, with 15.9% considered proactive and 84.1% reactive deprescribing.^15^ The majority of discontinuations (156; 78%) were not consistent with the definitions for proactive or reactive deprescribing. Although the population in this study^15^ were not end-of-life patients, the results in the present study reported similar proportions of proactive and reactive deprescribing.

End-of-life prognosis may lead to reactive deprescribing if a patient’s ability to take medication diminishes due to symptom deterioration or organ failure. For certain medications, time until benefit exceeds life expectancy, thus deprescribing could be proactive at the end of life. The present study examined prescribing for decedents in the last 2 weeks of life where death was imminent. Thus, end-of-life prognosis led to a reactive deprescribing. Guidelines and resources are needed to facilitate medication rationalisation and deprescribing for older people at the end of life. Intervention studies should be conducted to determine the impact of deprescribing on quality of prescribing in older people with life-limiting illness at the end of life.

### Strength and limitations

To our knowledge, this is the first study to differentiate between reactive and proactive deprescribing in older people receiving hospice care at the end of life. Some limitations need to be considered when interpreting the results. Firstly, the retrospective design, with medication prescription and discontinuation data retrospectively extracted from medication records, meant that there was a lack of information on some occasions such as the reason for medication discontinuation. Secondly, ‘when required’ medications were not included, and this may have resulted in an underestimation of the actual number of symptom control medications. Thirdly, the lack of access to patients’ files prior to hospice admission may have led to an underestimation of PPIs, H_2_ receptor antagonists, neuroleptic antipsychotics, long-term oral NSAIDs and long-term oral steroids using STOPPFrail, in which duration of use must be considered. Finally, the lack of availability of clinical data such as blood pressure, blood sugar and glycated haemoglobin may have resulted in overestimation of potentially inappropriate medications such as antihypertensives and antidiabetics according to STOPPFrail.

## Conclusions

Potentially inappropriate medications are commonly prescribed and continue to burden older people at the end of life. There was a clear difference in the way medication appropriateness criteria identify potentially inappropriate medications in older people at the end of life. In general, the criteria developed by Morin et al. identified more potentially inappropriate medications than STOPPFrail. The limited proportion of proactive deprescribing suggests limited adoption of pre-emptive deprescribing interventions in routine clinical practice.
